# Surface Exposure of PEG and Amines on Biodegradable Nanoparticles as a Strategy to Tune Their Interaction with Protein-Rich Biological Media

**DOI:** 10.3390/nano9101354

**Published:** 2019-09-20

**Authors:** Claudia Conte, Giovanni Dal Poggetto, Benjamin J. Swartzwelter, Diletta Esposito, Francesca Ungaro, Paola Laurienzo, Diana Boraschi, Fabiana Quaglia

**Affiliations:** 1 Department of Pharmacy, University of Napoli Federico II, Via D. Montesano 49, 80131 Napoli, Italy; claudia.conte@unina.it (C.C.); diletta.esposito@unina.it (D.E.); ungaro@unina.it (F.U.); 2 Institute for Polymers, Composites and Biomaterials, CNR, Via C. Flegrei 34, 80078 Pozzuoli (Napoli), Italy; giovanni.dalpoggetto@ipcb.cnr.it (G.D.P.); paola.laurienzo@ipcb.cnr.it (P.L.); 3 Institute of Biochemistry and Cell Biology, CNR, Via P. Castellino 111, 80131 Napoli, Italy; swartzwe@colorado.edu (B.J.S.); d.boraschi@ibp.cnr.it (D.B.); 4 Stazione Zoologica Anton Dohrn, Via A. Caracciolo 333, 80121 Napoli, Italy

**Keywords:** PEGylated nanoparticles, cationic nanoparticles, biological barriers, proteins

## Abstract

Nanoparticles (NPs) based on amphiphilic block copolymers of polyethylene glycol (PEG) and biodegradable polyesters are of particular current interest in drug nanodelivery due to their easily manipulated properties. The interaction of these NPs with biological environments is highly influenced by shell features, which drive biological identity after administration. To widen the strategies available for tuning particle surface chemistry, here we developed a panel of amine-bearing PEGylated NPs with a poly(ε-caprolactone) (PCL) core for the delivery of lipophilic drugs, and investigated the impact of NP modifications on their interaction with abundant circulating proteins (human serum albumin—HSA—and mucin), as well as their transport through biological barriers (artificial mucus—AM, extracellular matrix—ECM). We prepared NPs based on a diamino-terminated PCL (amine-NPs) and its mixture with PEG-PCL copolymers (amine/PEG-NPs) at different PEG molecular weights by nanoprecipitation, as well as corresponding NPs of PEG-PCL (PEG-NPs). The presence of an amine-bearing polymer resulted in NPs with a net positive charge and a zeta potential dependent on the length of PEG in the copolymer. Amine/PEG-NPs had a larger fixed aqueous layer thickness as compared to PEG-NPs, suggesting that PEG conformation is affected by the presence of positive charges. In general, amine-bearing NPs promptly interacted with the dysopsonic protein HSA, due to electrostatic interactions, and lose stability, thereby undergoing time-related aggregation. On the other hand, amine/PEG-NPs interaction with mucin induced switching to a negative surface charge but did not alter the quality of the dispersion. The transport kinetics of NPs through a layer of artificial mucus and tumor extracellular matrix was studied by means of fluorescent NPs based upon FRET. Amine/PEG-NPs did not cross the ECM, but they were promptly transported through the AM, with swifter transport noted at increasing MWs of PEG in the copolymer. Finally, we demonstrated that all the different NP types developed in this study are internalized by human monocytes and, despite the positive charge, they did not induce a measurable inflammatory effect. In conclusion, we showed that the concurrent presence of both PEG and amine groups on NP surface is a promising strategy for directing their interaction with body compartments. While PEG-NPs are confirmed for their capacity to cross ECM-like compartments, amine/PEG-NPs are revealed as a powerful platform to widen the arsenal of nanotools available for overcoming mucus-covered epithelia.

## 1. Introduction

Polyethylene glycol (PEG)-coated biodegradable nanoparticles (NPs) based on amphiphilic block copolymers are earning increased attention in the nanodelivery field due to their complete degradability in the body, ease of processing, and synthesis scalability. Owing to their core-shell structure, the hydrophobic inner compartment can host single or multiple drug(s), protects the payload from the harsh environment in the body, and ensures sustained release. On the other hand, the hydrophilic outer shell can be tailored toward deliberate interactions with the biological environment, besides providing superior physical stability to the entire manufacturing process [1,2]. The effects of PEGylation are closely related to the PEG molecular weight, the number of PEG chains located on NP surface, and the corresponding orientation (brush/mushroom), each of which can be affected by the preparation method [3,4].

For nanomedical approaches that imply intravenous NP administration, PEGylation is the most common strategy to extend their half-life, since the hydrophilic PEG fringe hinders adsorption of opsonin on NP surface, thus avoiding prompt recognition by the mononuclear phagocyte system [5,6]. PEGylation is also useful for improving NP transport through protein-rich gel barriers in the body [7,8]. Precise PEG physico-chemical properties and conformation on the particle surface are needed to effectively evade interaction with mucin and facilitate NP transport through mucus-covered epithelial barriers.

One of the drawbacks of PEGylated NPs is that cell internalization is impaired, which decreases the delivery of the drug payload to intracellular targets. To overcome poor cell uptake, the preeminent strategy is the decoration of NPs with ligands that target receptors on the cell surface, thereby allowing for internalization via receptor-mediated endocytosis [9]. Recently, we have explored the modification of the surface features of biodegradable PEGylated NPs made of poly(ε-caprolactone) (PCL) in view of their application as a drug delivery platform. The introduction of amine groups on the surface, which imparts a positive charge to NPs, could in fact become an additional tool to expand the possibility to manipulate NP properties and their interaction with the bioenvironment. Advantages of cationic NPs are mainly related to their unique ability to penetrate deeply inside tumor tissue [10,11] and bacterial biofilms, which could expand the arsenal of nanotools for the delivery of drugs through different routes [12,13]. Despite their potential, the strategies proposed thus far to build cationic NPs are limited, mainly relying on the adsorption of cationic surfactants, polymers, or phospholipids on the surface of preformed biodegradable nanotemplates [14]. Only recently, NPs prepared from ammonia-terminated [15] and amine-terminated biodegradable polymers have been reported [16]. Envisaging an application of amine-bearing NPs in cancer, we demonstrated that size, surface charge, shell thickness, and interaction with the human serum of these NPs were remarkably affected by the presence of PEG chains on the surface [16].

In terms of interactions with the biophase, it is expected that manipulation of the NP chemical identity would result in dramatic variations to its biological behaviour. In fact, interaction with the biological environment results in the formation of a biomolecular corona around NPs, which drives their in vivo behavior, cytotoxicity, immunotoxicity and activity [17,18]. Proteins are key players in this phenomenon and the presence/absence of protein–NP interactions should not be overlooked. For instance, the great majority of NPs tested in cell cultures comes in contact with a protein-rich medium (e.g., protein from fetal bovine serum-FBS), which can get adsorbed on their surface, thereby changing their properties. This aspect is often poorly addressed or ignored when interpreting biological data. Consequently, linking chemical identity to biological behavior becomes unreliable, making the design of NPs with a particular delivery requirement a challenging task.

To fully understand the interaction of the amine-functionalized NPs with biological systems, we have developed a panel of amine-functionalized PEGylated NPs with different PEG lengths, and assessed their properties with specific regard given to their surface features, their behavior in protein solutions, their ability to permeate through gel-like barriers (tumor extracellular matrix and artificial mucus), and finally both their uptake by human innate immune cells as well as capacity to induce an inflammatory reaction [19].

## 2. Materials and Methods

### 2.1. Materials

Monomethoxy-polyethylene glycol with Mn 1.0 kDa (mPEG1.0k, Nanocs Inc., New York, NY, USA) and Mn 2.0 kDa (mPEG2.0k, Sigma-Aldrich, Milan, Italy) were dehydrated by azeotropic distillation with dry toluene in a Dean-Stark trap. Stannous-(2-ethylhexanoate)_2_ (Sn(oct)_2_), triethylamine (TEA), tosyl chloride (TsCl), triphenylphosphine (PPh_3_), 1,4-butandiol, sodium azide and PEG_5K_-PCL_5K_ were purchased from Sigma-Aldrich (Milan, Italy). ε-caprolactone (CL) (Sigma-Aldrich, Milan, Italy) was distilled over CaH_2_ under vacuum. All solvents (analytical grade) were purchased from Sigma-Aldrich (Milan, Italy). N,N-dimethylformamide (DMF) and dichlorometane (DCM) were dried before use. Sodium chloride (NaCl), potassium chloride (KCl), calcium chloride (CaCl_2_), sodium acetate (NaH_3_C_2_O_2_), sodium bicarbonate (NaHCO_3_), sodium citrate dihydrate, magnesium chloride hexahydrate (MgCl_2_·6H_2_O), sodium sulfate (Na_2_SO_4_), disodium phosphate (Na_2_HPO_4_), poloxamer 188 (Pluronic^®^ F68), Human Serum Albumin (HSA), Type II porcine mucin, ECM gel from Engelbreth-Holm-Swarm murine sarcoma, diethylenetriaminepentaacetic acid (DPTA), RPMI 1640 amino acids solution, and egg yolk emulsion (microbiology) were purchased from Sigma-Aldrich (Milan, Italy). 3,3′-Dioctadecyloxacarbocyanine perchlorate (DiO) and 1,1′-Dioctadecyl-3,3,3′,3′-tetramethylindocarbocyanine perchlorate (DiL) were purchased from Thermo Fisher Scientific (Monza, Milan, Italy).

### 2.2. Polymer Synthesis

#### 2.2.1. Synthesis of mPEG-PCL Diblock Copolymers

The linear diblock copolymers were prepared by ring-opening polymerization (ROP) of CL at 120 °C for 24 h using mPEG (1.0, or 2.0 kDa) as an initiator and Sn(Oct)_2_ as a catalyst (20% by mol). CL/initiator molar ratio = 36. Copolymers were isolated through dissolution of the crude product in CHCl_3_, precipitation in n-hexane, and removal of solvent by vacuum. ^1^H NMR (CHCl_3_, δ in ppm), PCL block: 1.29–1.78 (m), 2.19–2.43 (t) 3.20 (m); 3.92–4.21 (t), 4.31(t); PEG block: 4.10 (t), 3.64 (s), 3.38 (t).

#### 2.2.2. Synthesis of Diamine-PCL (NH_2_-PCL_4k_-NH_2_)

Step 1. Synthesis of PCL diol (HO-PCL4k-OH). 1,4-butandiol (500 mg, 5.55 mmol), CL (22.49 g, 197 mmol), and SnOct_2_ (449 mg, 1.1 mmol) were added in a flask under dry nitrogen. The polymerization was carried out under stirring at 120 °C for 24 h. The product was dissolved in 20 mL of DCM and precipitated in cold hexane; the polymer was collected and dried under vacuum. ^1^H NMR (CHCl_3_, δ in ppm): 1.29–1.78 (123H, m), 2.19–2.43 (74H, m), 3.92–4.21 (74H, t), 3.64 (4H, t) (Mn evaluated by ^1^H NMR = 4344 dalton).

Step 2. Synthesis of ditosyl-PCL (Ts-PCL_4k_-Ts). HO-PCL_4k_-OH (4.0 g, 0.92 mmol) was dissolved in 40 mL of DCM, then TEA (506 mg, 5.0 mmol) and TsCl (953 mg, 5.0 mmol) were added. The reaction was carried out under stirring at room temperature for 24 h. The polymer was dissolved in 15 mL of DCM and precipitated in cold hexane, then collected and dried under vacuum. ^1^H NMR (CHCl_3_, δ in ppm): 1.29–1.78 (123H, m), 2.19–2.43 (74H, m), 3.92–4.21(74H, t), 7.79 (4H, d), 7.49 (4H, d), 2.43 (6H, s).

Step 3. Synthesis of diazide-PCL (N_3_-PCL_4k_-N_3_). Ts-PCL_4k_-Ts (1.0 g, 0.66 mmol) was dissolved in 15 mL of dry DMF, then NaN_3_ (214 mg, 3.3 mmol) was added and the mixture was stirred overnight at 90 °C under nitrogen stream. The reaction mixture was then cooled down to room temperature and filtered, and DMF was removed under vacuum. The crude product was dissolved in 10 mL of DCM and washed twice with brine and twice with water in a separating funnel. The organic phase was dried over anhydrous Na_2_SO_4_, concentrated, poured into cold hexane, and the precipitated polymer was dried under vacuum. FTIR diagnostic band: 2107 cm^−1^ (N_3_ stretching) ^1^H NMR (CHCl_3_, δ in ppm): 1.29–1.78, (123H, m), 2.19–2.43, (74H, m); 3.92–4.21(74H, t).

Step 4. Synthesis of diamine-PCL (NH_2_-PCL_4k_-NH_2_). N_3_-PCL_4k_-N_3_ (2.0 g) was dissolved in 50 mL of MeOH at 40 °C in a flask equipped with a nitrogen inlet and refrigerator, then PPh_3_ (N_3_/PPh_3_ molar ratio = 1/3) was added and the reaction was carried out at 100 °C overnight. After removing the solvent by rotary evaporation, the polymer was dissolved in 10 mL of DCM, precipitated in cold hexane, collected, and finally dried under vacuum. The occurrence of the reaction was confirmed by FTIR through disappearance of the 2097 cm^−1^ N_3_ stretching band. ^1^H NMR (CDCl_3_, δ in ppm): 1.29–1.78, (123H, m), 2.19–2.43, (74H, m); 3.92–4.21(74H, t), 3.64 (4H, t), 2.48 (4H, broad). GPC analysis: Mw 4.8 kDa; Mn 4.2 kDa; Mw/Mn 1.1.

### 2.3. Polymer Characterization

FTIR analysis was performed with a Perkin-Elmer spectrometer (Paragon 500, Norwalk, CT, USA) equipped with a ZnSe attenuated total reflectance (ATR) crystal accessory. ^1^H NMR spectra were recorded with a Bruker Avance DPX400 apparatus (Billerica, MA, USA) operating at 400 MHz at 25 °C and 128 NS. GPC analysis was performed using a Malvern-Viscotek GPC-MAX/TDA 305 quadruple detector array (Malvern, UK) equipped with a precolumn and two Phenogel columns (Phenomenex, Torrance, CA, USA) with exclusion limits of 10^6^ and 10^3^, respectively. THF solutions (100 μL) were filtered (PTFE 0.22 μm) and analyzed at a flow rate of 0.8 mL/min and a temperature of 35 °C. Calibration was based on a standard of polystyrene (Mw 104,959 Da).

### 2.4. Preparation and Characterization of Nanoparticles

PEGylated and amine/PEGylated NPs were prepared by the solvent-diffusion method. In particular, we prepared PEG-NPs series (PEG_1K_-PCL_4K_, PEG_2K_-PCL_4K_, and PEG_5K_-PCL_5K_), amine-NPs (NH_2_-PCL_4k_-NH_2_), and amine/PEG-NPs (NH_2_-PCL_4k_-NH_2_ mixed with either PEG_1K_-PCL_4K_ or PEG_2K_-PCL_4K_ or PEG_5K_-PCL_5K_ at 1:1 w/w ratio). Briefly, 10 mg of polymers were dissolved in 2 mL of acetone and added dropwise in 4 mL of water containing 4 mg of Pluronic® F68 as surfactant (0.1% w/v) under stirring. Acetone was removed under vacuum (5 min). Finally, the NP dispersion was transferred and stored in Eppendorf tubes at a final NP concentration of 2.5 mg/mL. NPs were tested shortly after preparation.

Fluorescent NPs giving FRET (FRET-NPs) were prepared by nanoprecipitation as described above with minor modifications. DiO (λ_ex_ = 488 nm, λ_em_ = 505 nm) and DiL (λ_ex_ = 543 nm, λ_em_ = 575 nm) were selected as FRET pair. Briefly, 100 μL from each DCM stock solution of DiO and DiL (1 mg/mL) were transferred to a clean vial and left to evaporate for 30 min until a colorless film was formed. Then, the copolymer solution in acetone was added. Nanoprecipitation was carried out as reported above. After preparation, NPs were filtered (RC 0.45 μm) to remove any free dye molecules. DiL and DiO loading inside NPs was assessed by dissolving 1 mg of freeze-dried NPs in 1 mL of DCM under stirring for 1 h. Samples were analyzed for DiO and DiL quantification by UV spectrophotometry at 488 and 543 nm, respectively. The concentration of DiL and/or DiO was calculated by means of a standard calibration curve derived for DCM solutions of the specific dye at known concentrations (0.5–60 μg/mL). Potential interference from DiL on DiO absorbance and vice versa were assessed by spiking a DiO solution in DCM with different amounts of DiL, or a DiL solution in DCM with different amount of DiO. To verify a possible interference of copolymers on DiL/DiO quantitative analysis, a weighted amount of unloaded NPs was dissolved in DCM and analyzed under the same conditions reported for the dyes.

The hydrodynamic diameter (D_H_), polydispersity index (PI), and zeta potential (ζ) of NPs were determined on a Zetasizer Nano ZS (Malvern Instruments Ltd.). Results are reported as the mean of three separate measurements of three different batches (n = 9) ± standard deviation (SD).

Pluronic^®^ F68 associated to NPs was assessed by quantitative ^1^H NMR. After preparation, NPs were centrifuged at 2300× *g* for 20 min and the supernatant containing unabsorbed surfactant was lyophilized. The obtained solid was dissolved in D_2_O and analyzed by ^1^H NMR. The quantitative determination of Pluronic^®^ F68 was achieved by comparing the integral of –CH_3_ protons at 1.23 ppm in the samples with the corresponding signal of a spectrum of pure Pluronic® F68 of a known concentration (2 mg/mL). The amount of Pluronic^®^ F68 absorbed on the NP surface was determined indirectly by the difference between the initial amount of Pluronic® F68 (4 mg) and the calculated amount of residual Pluronic^®^ F68 in solution. 

Stability studies of NPs as prepared (0.5 mg/mL) were conducted over a 24-h time span by monitoring size, ζ, and scattering (absorbance at 500 nm on a UV-1800 spectrophotometer, Shimadzu Corporation, Tokyo, Japan).

### 2.5. Evaluation of NP Surface Features

Fixed aqueous layer thickness (FALT) measurements were based on the approximation of the Gouy–Chapman theory and carried out by monitoring the influence of ionic strength on the particle surface [20,21]. Different amounts of NaCl stock solutions at different concentrations were added to an NP dispersion in water (0.5 mg/mL), and ζ of the samples was measured. A plot of ln (ζ) against 3.33·[NaCl]^0.5^ results in a straight line, where the slope represents the thickness of the PEG shell in nm.

The amount of PEG on the NP surface was evaluated through ^1^H NMR. Spectra were recorded for either NPs dispersed in D_2_O (5 mg/mL) or dissolved in CDCl_3_ (5 mg/mL). The amount was calculated by comparing the integral of the -CH_2_- resonance at 3.6 ppm of PEG in D_2_O with the corresponding signal in CDCl_3._ The -CH_2_- integral relative to PEG was calculated considering the contribution of -CH_2_- from Pluronic^®^ F68:

$$\mathrm{int}.{\;\mathrm{CH}}_{2}{}_{\left(\mathrm{PEG}\right)}=\mathrm{int}.{\mathrm{CH}}_{2}{}_{\left(\mathrm{total}\right)}-8\times \mathrm{int}.{\mathrm{CH}}_{2}{}_{\left(\mathrm{Plur}\right)}$$

where 8 × int.CH_3(Plur)_ was obtained from a pure Pluronic® F68 spectrum (ratio -CH_3_/CH_2_ = 1/8).

### 2.6. Interactions with Proteins

Interaction of NPs with HSA or mucin was assessed by fluorescence spectroscopy, DLS measurements and turbidimetry analyses. For interaction with HSA, 200 μL of NPs (2.5 mg/mL) were mixed with 100 μL of HSA stock solution in water (2 mg/mL) and 700 μL of water. The final concentrations of NPs and HSA in the samples were 500 and 200 μg/mL, respectively. For interaction with mucin, mucin powder was dispersed in water (0.16% w/v) and stirred overnight. Then, the dispersion was centrifuged at 2300× *g* for 20 min and the supernatant collected. Thereafter, 200 μL of NPs were mixed with 250 μL of mucin dispersion and water up to a final volume of 1 mL. The final concentrations of NPs and mucin in these samples were 500 μg/mL and 0.04% w/v, respectively [22]. Control samples of HSA, mucin, and NPs were run as a control.

Fluorescence spectroscopy was used to assess the ‘quenching’ effect of NPs on the ability of certain residues of the protein to emit light. Following preparation, the samples were incubated at RT for 1 h. Then, the emission spectra were acquired (Ex = 278 nm) (RF-6000, Shimadzu Corporation, Tokyo, Japan) [3]. At different time points (0, 4, and 24 h), size, ζ potential, and scattering were measured as described above.

### 2.7. Permeation of NPs through Gel-Like Barriers

The capacity of NPs to cross artificial mucus (AM) and a tumor ECM was investigated. The transport experiment was carried out by placing either artificial mucus (AM) (for composition, see SI) or ECM gel (from a murine sarcoma) in the upper chambers of Transwell^®^-12 well plates (12 mm diameter, polyester membranes with a 3.0 μm pore size), applying NPs, and monitoring the amount of NPs that diffused into the lower chamber [23]. For transport through the AM, the lower chamber was filled with 1 mL of simulated interstitial lung fluid (SILF) (for composition, see Supplementary Information (SI)). For transport through the ECM gel, the lower chamber was filled with 1 mL of 0.01 M PBS (phosphate buffer saline, NaCl 0.138 M, KCl 0.0027 M), pH 7.4 at 25 °C. FRET-NPs (0.1 mL, 2.5 mg/mL) were placed on the top of gel layer (0.3 mL) and maintained at RT. At 0, 1, 4, and 24 h, the medium in the lower chamber was collected and the fluorescence emission spectra recorded at λ_Ex_ = 488 and 543 nm to determine the FRET efficiency ratio and the NP concentration, respectively.

To calculate the NP concentration in SILF and PBS, a calibration curve of DiL emission intensity at Ex = 543 nm against different known concentrations of NPs was generated for each formulation. FRET efficiency was calculated for DiO λ_ex_ = 488 nm emission spectra using the following equation [24]:

$$\mathrm{FRET}\;\mathrm{efficiency}\;=\;\frac{\mathrm{Intensity}\;\mathrm{at}\;\mathrm{Em}=575\;\mathrm{nm}}{\mathrm{Intensity}\;\mathrm{at}\;\mathrm{Em}\;=\;575\;\mathrm{nm}\;+\;\mathrm{Intensity}\;\mathrm{at}\;\mathrm{Em}\;=\;505\;\mathrm{nm}}$$

The stability of FRET-NPs in SILF and PBS was monitored until 24 h through the DLS measurements.

### 2.8. NP Interaction with Human Immune Cells

#### 2.8.1. Monocyte Isolation

Human primary blood monocytes were isolated from healthy donors with informed consent. Briefly, peripheral blood mononuclear cells were separated from freshly obtained whole blood by gradient density centrifugation on Ficoll-Paque PLUS (GE healthcare, Bio-Sciences AB, Uppsala, Sweden). CD14 receptor-positive monocytes were further isolated by magnetic cell sorting using CD14 microbeads following the manufacturer’s protocol (Miltenyi Biotec, Bergisch-Galdbach, Germany). Cell viability was assessed by trypan blue dye exclusion and determined to be >98%.

#### 2.8.2. NP Uptake

Freshly isolated CD14+ monocytes were seeded in 24-well plates (Corning^®^ Costar^®^; Corning Inc. Life Sciences, Oneonta, NY, USA) at 2 × 10^5^ cells/well in 0.5 mL of Phenol Red-free RPMI 1640 medium (GIBCO by Life Technologies, Paisley, UK) supplemented with 5% heat-inactivated pooled human AB serum (Sigma-Aldrich) and 50 μg/mL gentamicin sulfate (GIBCO). After 24 h at 37 °C, the cells were washed and exposed for 2 h to 50 μg/mL of DIL-loaded NPs (NPs pre-treated for 1 h with 70% HSA). The extracellular media were then collected and the fluorescence intensity of DiL was measured at λ_ex/em_ = 543/575 nm in a microplate reader (Cytation 3 imaging reader, Biotek, Winooski, VT, USA). The concentration of NPs in the extracellular compartment was calculated by means of a standard calibration curve derived for NP dispersions in the cell medium at known concentrations (0.5–50 μg/mL). For confocal microscopy, monocytes were seeded on glass cover slips and treated as described above. Following NP incubation, cells were fixed for 20 min in 3.7% formaldehyde, washed 3 times with PBS, and cover slips were mounted on slides with Vectashield mounting medium containing 4′,6-diamidino-2-phenylindole (Vector Laboratories Inc., Burlingame, CA, USA). Confocal microscopy was conducted using a Zeiss LSM 700 confocal microscope.

#### 2.8.3. Monocyte Activation

Isolated monocytes were seeded in 24-well culture plates at 5 × 10^5^ cells/well in 1 mL of RPMI 1640 medium supplemented with 5% heat-inactivated human AB serum and 50 μg/mL gentamicin sulfate. Cells were exposed to NPs (pre-treated for 1 h with 10% human AB serum) ±1 ng/mL LPS from *Escherichia coli* (*E. coli*, serotype O55:B5, Sigma-Aldrich) for 1 h. The concentration of NPs incubated in each well was calculated based upon the NP surface area as indicated in Table S1. After 24 h of incubation, supernatants were collected and frozen at −20 °C until use. Activation was assessed as release in the supernatant of the inflammatory/defensive cytokines Interleukin-1 beta (IL-1β) and Tumor Necrosis Factor alpha (TNFα), measured by ELISA with commercially available kits (DuoSet ELISA, R&D Systems, Minneapolis, MN, USA) following the manufacturer’s instructions.

## 3. Results and Discussion

### 3.1. Synthesis and Characterization of the Polymers

**Figure 1:**
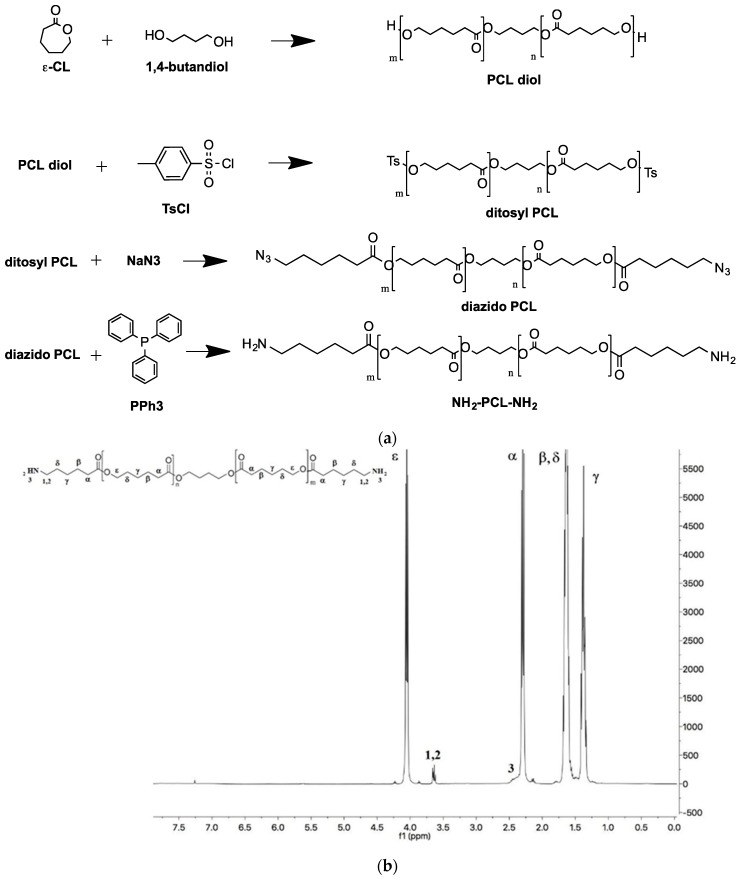
(**a**) Steps for the synthesis of NH_2_-PCL-NH_2_. (**b**) ^1^H NMR spectrum of NH_2_-PCL-NH_2_ in CDCl_3_.

The mPEG-PCL diblock copolymers were synthesized by classical ROP polymerization, using mPEG-OH of different molecular weights as an initiator. Theoretical molecular weights were in agreement with the values found by GPC and determined by ^1^H NMR (Mn of PCL block was calculated from the ratio between intensities of the resonance associated to -O–CH_3_ protons of mPEG end groups at 3.36 ppm and –CH_2_–CO– units in the PCL chain at 2.31 ppm) (Table S2). Diamine-PCL (H_2_N-PCL-NH_2_) was synthesized according to the scheme reported in Figure 1a.

Full conversion of azide end-groups of N_3_-PCL-N_3_ precursor to amines was checked by FTIR through disappearance of the azide band at around 2100 cm^−1^ (Figure S1). The structure of the final polymer was confirmed by ^1^H -NMR as reported in Figure 1b.

### 3.2. Nanoparticle Properties

**Figure 2:**
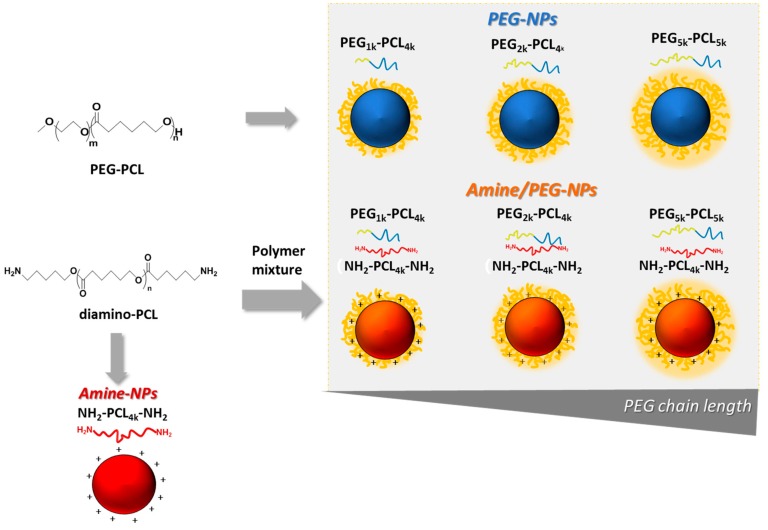
Schematic representation of nanoparticles tested in the study.

A panel of core-shell NPs was obtained from 1:1 mixtures of NH_2_-PCL-NH_2_ and PEG-PCL (amine/PEG-NPs), and single NH_2_-PCL-NH_2_ (Amine-NPs) or PEG-PCL (PEG-NPs) mixtures as shown in Figure 2. PEGs of different molecular weights were employed in the study (1, 2, 5 kDa) while PCL moiety remained fixed (~4 kDa).

Pluronic^®^ F68 was needed to fabricate amine-bearing NPs with good yields yet avoiding their premature aggregation. For comparison purposes, its use was extended to all the formulations tested. Indeed, poloxamer was associated to all the types of PEGylated NPs and its surface amount decreased as PEG MW increased (Table 1). NPs had an average size below 150 nm and a monomodal distribution, a finding also supported by the low PI values (Table 1). The increase of PEG MW in the copolymer decreased D_H_ for both PEG-NPs and amine/PEG-NPs. A positive ζ was observed in the amine-bearing variants, which was decreased in the amine/PEG series, especially at increasing PEG MW. As expected, PEG-NPs had a negative ζ.

The short-term stability of NPs in water was observed up to 24 h by monitoring scattering, D_H_, and ζ (Figure S2a,b). Scattering analysis of NPs suggested that no aggregation occurred over 4 h due to negligible changes in absorbance values at 500 nm and ζ. Amine-NPs and Am/PEG_1K_-PCL_4K_ NPs showed an increase of D_H_ values and large fluctuations of ζ after 24 h while the other NPs showed no significant differences in D_H_. Size distribution curves in water (Figure S2c) clearly highlight that PEG_1K_ is not able to confer satisfactory colloidal stability to Am/PEG_1k_-NPs in analogy to PEG_1k_-NPs. Overall, these results indicate that NPs should be tested within 4 h of preparation. From a clinical perspective, freeze-drying of the NPs dispersion, likely in the presence of a cryoprotectant, is needed to ensure suitable shelf-life and storage stability.

### 3.3. Extent of Nanoparticle PEGylation

**Figure 3:**
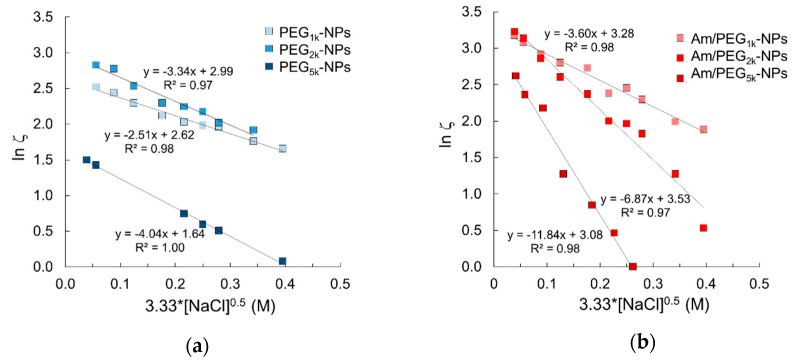
Fixed aqueous layer thickness (FALT) measurements for (**a**) PEG-NPs and (**b**) Am/PEG-NPs.

Fixed aqueous layer thickness (FALT) of the outer shell of NPs was determined by measuring ζ as a function of the sodium chloride concentration at pH values of 7.0 where primary amines are expected to be ionized. As reported in Figure 3, the slope value of the linear regression line obtained plotting log ζ vs sodium chloride concentration gives the shell thickness (nm) for each NPs type. As expected, shell thickness increased as PEG MW increased. Interestingly, Am-PEG-NPs displayed thicker aqueous layers as compared with the corresponding PEG-NPs counterparts (11.8 nm for Am/PEG_5K_-NPs vs. 4.0 nm for PEG_5K_-NPs). The shell thickness for amine/PEG-NPs increased with respect to the increasing PEG chain length, as was the case for the PEG-NPs. However, the increase across the series was greatest for the amine/PEG-NPs. The extent of the shell thickness increase for amine/PEG-NPs was steeper and more profound.

The amount of PEG on the surface of NPs was assessed by quantitative ^1^H NMR (Table 2). As can be seen, NPs exposed much less PEG than expected, suggesting that during nanoprecipitation, PEG chains are entangled in the PCL core. This result confirms that the preparation method has a major impact on the PEGylation extent, and also on the conformation of the flexible hydrophilic cloud on the NPs surface.

### 3.4. Nanoparticle Interactions with Proteins in Solution

Physical properties of NPs, such as size, shape and surface composition, strongly affect the interaction with proteins in complex media, which can alter the chemical identity of NPs and in turn their biological fate. HSA is a dysopsonic protein that can extend NP circulation time [25] and its mode of interaction with NPs can impact cell uptake [26]. On the other hand, mucin is a barrier protein that is relevant in the case of NP interactions with mucosal surfaces. Thus, we studied HSA and mucin interaction with the panel of NPs fabricated through the combination of fluorescence spectroscopy, turbidimetry and size measurements.

Protein adsorption onto the nanoparticles, which can affect their stability over time, was monitored by measuring the absorbance value (at λ = 500 nm) of NPs dispersions in the presence of HSA and mucin, over time (Figure S3). As the proteins absorb onto the NPs, light scattering occurs, which changes the absorbance value over time. As can be seen in Figure S3, only Am-NPs suffered from stability issues in the presence of proteins, showing a tendency to increase the scattering of light over time, whereas no significant effect was found for the PEGylated series.

**Figure 4:**
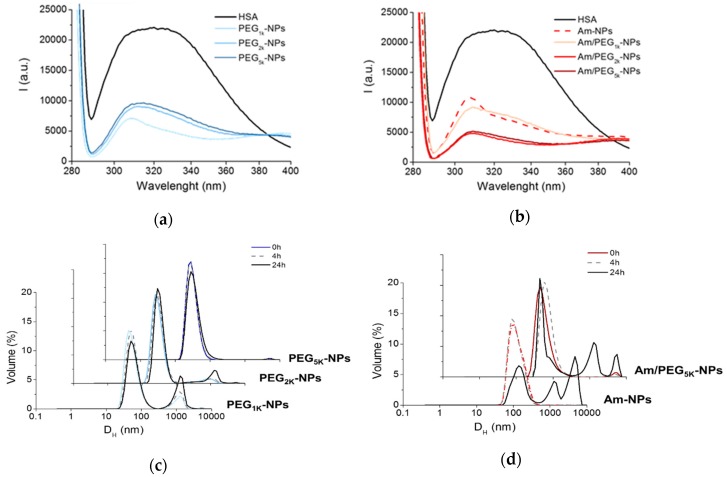
Interaction of NPs with proteins. Fluorescence emission spectra of HSA (0.2 mg/mL) at Ex = 278 nm in the presence of PEG-NPs (**a**) and Am-NPs or Am/PEG-NPs (**b**) (NPs = 0.5 mg/mL). Spectrum of free HSA is reported as control. Representative size distribution curves of PEG-NPs (**c**) and Am-NPs or Am/PEG-NPs (**d**) incubated in HSA (0.2 mg/mL) (NPs = 0.5 mg/mL).

Figure 4a,b show the fluorescence emission spectra of HSA in the absence and in the presence of NPs upon excitation at 278 nm. The black spectrum shows the typical dual band fluorescence spectrum of HSA, which reflects the contribution of the tyrosine (λ_em_ ca 310 nm) and tryptophan (λ_em_ ca 340 nm) fluorogenic centers. This strong emission is quenched upon addition of PEG-PCL NPs simply due to static quenching effects arising by the massive aggregation of HSA on the NPs [3]. This result suggests that: (i) A PEG shell is unable to prevent NP–protein hydrophobic interactions; (ii) Am-NPs adsorb HSA, presumably due to electrostatic interactions, and (iii) amine-PEG NPs interact with HSA through combined hydrophobic/electrostatic interaction. These results are in line with those found for a series of PEGylated cationic liposomes that adsorbed human plasma proteins depending on PEG length [27].

Particle size in the presence of proteins was monitored over time using DLS, as shown in Figure 4c,d. PEG-NPs experienced a high degree of protection from aggregation, especially for the PEG_5k_ variant (Figure 4c). On the contrary, amine-bearing NPs showed a completely different behavior, exhibiting a fast and time-dependent aggregation that even a PEG_5k_ copolymer was unable to hamper. These data demonstrate that PEGylated NPs are only partly capable of shielding HSA interaction, presumably due to the low amount of surface PEG and marginal effect of the adsorbed poloxamer.

As far as mucin interaction is concerned, its interaction with cationic NPs has been routinely used to achieve mucoadhesion [28]. Nevertheless, precise tuning of PEG grafting density and molecular weight is considered a strategic approach to minimize mucoadhesion and in turn promote NPs transport through a mucus layer [7].

**Figure 5:**
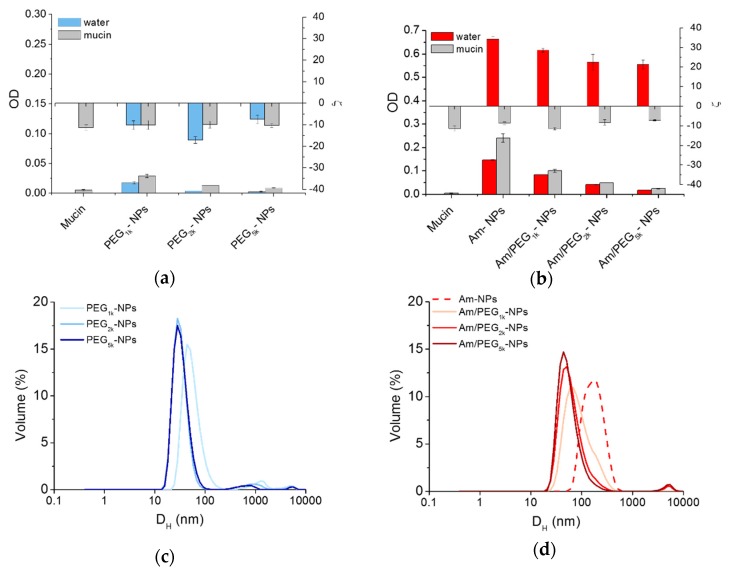
Interaction of NPs with mucin. Scattering of PEG-NPs (**a**) or Am-NPs and Am/PEG-NPs (**b**) in water or in a mucin water dispersion (0.08% w/v) (NPs = 0.5 mg/mL). Data are an average of triplicate measurements ± SD. Representative size distribution curves of PEG-NPs (**c**) or Am-NPs and Am/PEG-NPs (**d**) in a mucin water dispersion (0.08% w/v) (NPs = 0.5 mg/mL).

Mucoadhesive behavior of NPs can be estimated on the basis of an increased absorbance at 500 nm [29], while interactions between mucin and positively charged NPs can be monitored by size measurements [8]. There was little quenching of mucin fluorescence in the presence of all NPs (Figure S4), suggesting that mucin was not significantly interacting with the NPs. As shown in Figure 5a, scattering in water increased only for Am-NPs, suggesting that such an interaction with the protein occurs. Nevertheless, ζ was unchanged for PEG-NPs and switched to negative values for all amine-bearing NPs. These results indicate that mucin negative chains adsorb onto positive Am-NPs via electrostatic interactions between opposite charges, which are not shielded by the concurrent presence of PEG chains. As evidenced by the corresponding size curves in Figure 5c,d, the increase of PEG length is increasingly effective in ensuring satisfactory stability while avoiding interactions between NPs. Thus, PEG length and its surface conformation remains the main determinant for regulating behavior at the interface also for amine-modified NPs [8].

### 3.5. Permeation through Protein-Rich Gels

To ascertain if the developed NPs permeated differently through protein-rich gel-like barriers, we focused on a tumor ECM, which could mimic tumor stroma, and artificial mucus, which is representative of the bronchial secretion.

**Figure 6:**
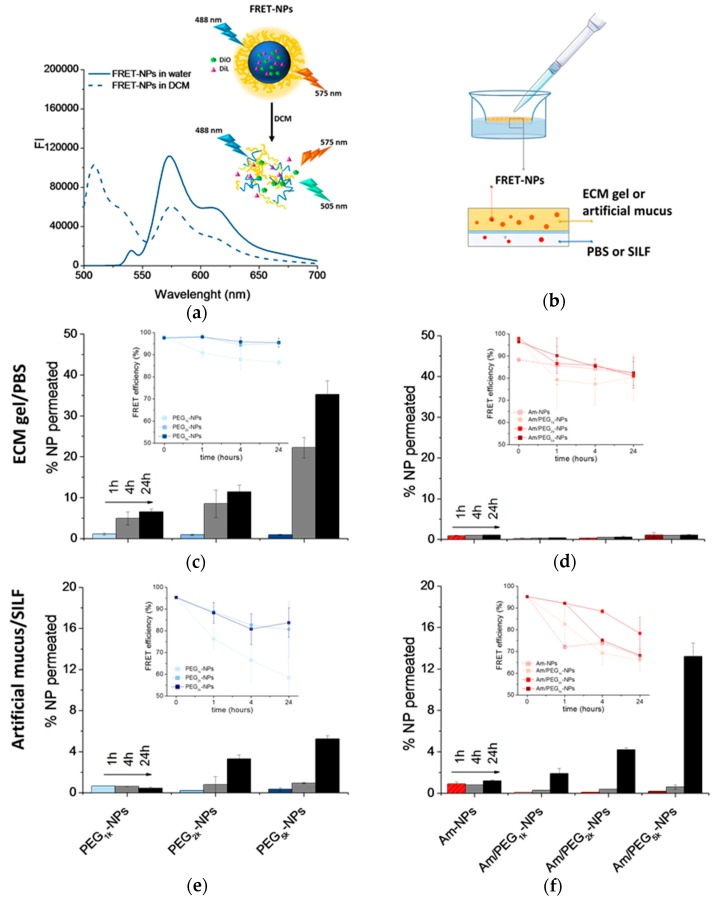
Permeation of NPs through protein-rich gels over time. For this experiment, NPs were loaded with DiO/DiL as a FRET pair. (**a**) Emission spectra collected after DiO excitation (λ_ex_ = 488 nm) of DiO/DiL-loaded PEG_5k-_NPs dispersed in water or dissolved in DCM. (**b**) Set-up of the transport experiment. Amount of DiO/DiL-loaded PEG-NPs (**c**,**e**) and amine-NPs and amine/PEG-NPs (**d**,**f**) found in the acceptor chamber after crossing the ECM gel (**c**,**d**) and artificial mucus (**e**,**f**). The amount of NPs in the acceptor medium was evaluated by monitoring DiL emission (λ_ex_ = 543 nm). In the inset, FRET efficiency is reported (for the calculation, see Section 2.7). Data are an average of triplicate measurements ± SD.

For this experiment, FRET NPs were developed. FRET is a mechanism describing energy transfer between two light-sensitive molecules, a donor chromophore and an acceptor. The efficiency of this energy transfer is inversely proportional to the sixth power of the distance between the donor and acceptor, making FRET extremely sensitive to small changes in distance. Measurement of FRET efficiency is a useful tool for determining if two fluorophores are released from an NP system and to monitor its disassembly in different biological conditions, in vitro and in vivo [23,30]. As donor and acceptor chromophores, we selected the hydrophobic dyes DiO (λ_ex/em_ is 488/505 nm) and DiL (λ_ex/em_ at 543/575 nm), respectively, since the DiO emission spectrum overlaps well with the DiL absorption spectrum. Figure 6a reports the emission spectra of PEG_5k_-NPs loaded with DiO/DiL as a representative FRET formulation in both water and DCM. Upon excitation of NPs in water at 488 nm, DiO emission at 505 nm is quenched due to FRET, and DiL emission is enhanced. When NPs are dissolved in DCM, DiO emission is restored and DiL emission is depressed since no FRET occurs. Mean particle size was doubled only in FRET-based PEG-NPs while ζ of all NPs types were comparable to their unloaded counterparts (cfr D_H_ and ζ in Table 1 and Table S3). NP stability and constant FRET efficiency in the acceptor medium was demonstrated for all NP formulations (Figure S5); a fundamental prerequisite for the validation of results in transport experiments.

The percentage of NPs permeated through the ECM or mucin over time was evaluated according to the set-up described in Figure 6b. At different time points, the NP amounts in the acceptor chamber were evaluated by measuring DiL emission (λ_ex_ = 543 nm). As reported in Figure 6c, PEG-NPs showed a time-dependent transport through the ECM gel, with the extent of permeation clearly increasing as PEG MW increased. On the contrary, Am-NPs and Am/PEG-NPs did not show any capability to permeate through the ECM gel (Figure 6d). In the case of artificial mucus, we found that increasing PEG MW allowed an increase of NP penetration for both PEG-NPs (Figure 6d), and very surprisingly, this was also the case for Am/PEG-NPs (Figure 6e). In fact, the amount of transported Am/PEG_5k_-NPs was higher than that corresponding to PEG_5k_-NPs, highlighting that the presence of amine groups coupled with long PEG chains could even facilitate transport. Nevertheless, independently of the PEG MW, Am/PEG-NPs did not show any aggregation in SILF over time, as confirmed by DLS measurements (Figure S6). It is worth noting that permeation of Am-NPs was affected by their poor stability in both PBS (Figure S5) and SILF (Figure S6).

Emission spectra of DiL collected at DiO excitation (Figure S7) allowed calculation of the FRET efficiency according to the equation reported in Section 2.7 (insets in Figure 6c–e). A FRET efficiency higher than 80% for all the samples at different time points demonstrated that the fluorescence emission is due to intact fluorescent NPs and that only a limited contribution from FRET pair leaching occurred.

### 3.6. Uptake and Immune Activation

The innate immune system is the first to come in contact with NPs entering the body. For this reason, we investigated both the uptake and immunostimulatory capacity of our synthesized NPs in human primary monocytes, which are the main innate immune cells.

**Figure 7:**
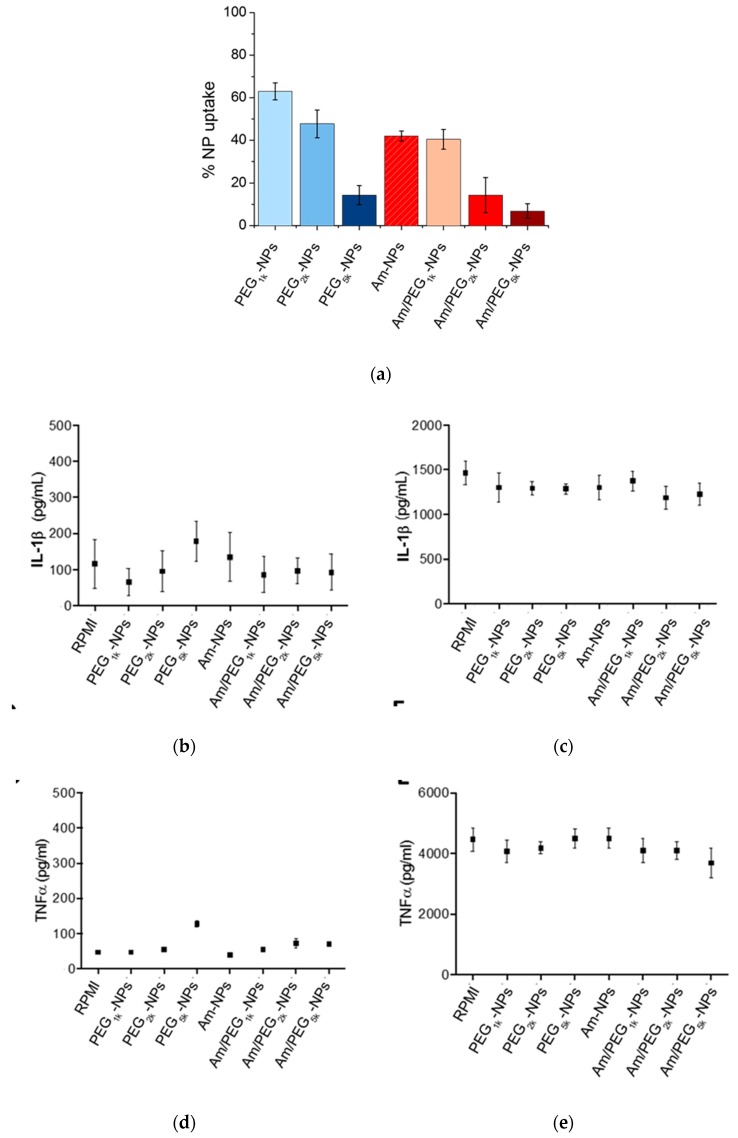
Uptake and inflammatory response to NPs by human monocytes. (**a**) Quantification of NPs taken up by monocytes after 2 h incubation. Production of IL-1β (**b**,**c**) and TNFα (**d**,**e**) by human monocytes after 24 h of incubation with NPs as assessed by an ELISA kit. Panels (**c**,**e**) refer to LPS stimulated monocytes. Results represent mean ± SEM, *n* = 2.

The extent of NP uptake by human monocytes was assessed by measuring the fluorescence of DiL in the extracellular compartment of cells, after exposing them to NPs for 2 h. The extent of NP uptake by human monocytes (Figure 7a and Figure S8) was roughly related to PEG length in the copolymer (greater PEG length results in lower uptake) following a very general rule for PEGylated systems, while the presence of amine groups on NPs surface had no impact.

The production of IL-1β and TNFα, which are indicators of inflammatory effects, was assessed using a commercially available ELISA kit following 24 h of NP incubation (Figure 7b,d). In no cases were the levels of IL-1β or TNFα markedly elevated or suppressed compared to background cytokine production. To assess whether the NPs may modulate induced innate immune responses, cells were additionally stimulated with the gram-negative bacterial molecule lipopolysaccharide (LPS), in the presence and absence of each NP formulation (Figure 7c,e). LPS stimulation resulted in elevated production of IL-1β and TNFα, a phenomenon that was not altered toward either increased or suppressed production in the presence of the NPs [19].

## 4. Conclusions

In this work, we prepared a panel of amine-functionalized PEGylated NPs (amine/PEG-NPs) with different PEG-chain lengths and evaluated their surface properties compared to corresponding PEG-NPs. The interaction of the NPs with human proteins, such as serum albumin and mucin, was studied using fluorescence spectroscopy, DLS, and turbidimetry analysis. The results demonstrated that amine-bearing NPs interacted strongly with proteins, and that this was found to be dependent on PEG length and surface charge. Furthermore, differences in their ability to permeate through protein-rich biological barriers were also found. Amine/PEG-NPs were unable to cross the ECM but were able to be transported through mucus, with transport facilitated by increasing MWs of PEG. Finally, the interaction of the different NP types with human innate immune cells (primary blood monocytes) was also studied. Cell uptake was found to decrease with increasing PEG MW and overall surface charge. Independently of uptake, NPs not were found to induce an inflammatory response, nor were they observed to interfere with a normal defensive reaction to bacterial agents, thereby demonstrating high tolerability. In conclusion, we demonstrated that PEGylation extent and the presence of amine groups on the NP surface are key elements that affect NP behavior in biological environments.

## Figures and Tables

**Table 1:** Colloidal properties of NPs. Values are expressed as mean ± SD of three different batches.

	Code	Composition	Yield (%)	Poloxamer ^2^ (mg)	D_H_^3^ (nm ± SD)	PI ^3^	ζ ^4^ (mV ± SD)
PEG-NPs	PEG_1K_-NPs	PEG_1K_-PCL_4K_	80	2.2	78 ± 0.3	0.224	−10.2 ± 2.0
	PEG_2K_-NPs	PEG_2K_-PCL_4K_	95	1.3	44 ± 1.3	0.160	−17.1 ± 1.6
	PEG_5K_-NPs	PEG_5K_-PCL_5K_	98	1.9	44 ± 3.6	0.175	−7.4 ± 1.9
Amine-NPs	Am-NPs	NH_2_-PCL_4K_-NH_2_	82	-	134 ± 0.3	0.128	34.3 ± 1.3
Amine/PEG-NPs ^1^	Am/PEG_1K_-NPs	NH_2_-PCL_4K_-NH_2k_ PEG_1K_-PCL_4K_	93	2.4	121 ± 2.8	0.191	28.6 ± 1.0
	Am/PEG_2K_-NPs	NH_2_-PCL_4K_-NH_2_ PEG_2K_-PCL_4K_	95	1.0	99 ± 5.9	0.257	20.0 ± 2.3
	Am/PEG_5K_-NPs	NH_2_-PCL_4K_-NH_2_ PEG_5K_-PCL_5K_	98	1.2	94 ± 6.8	0.250	21.3 ± 2.4

^1^: Copolymer mixture was 1:1 by wt.; ^2^: Pluronic^®^ F68 associated to NPs was derived from the amount found in the medium after NPs preparation. Quantitative ^1^H NMR measurements were taken as described in 2.4.; ^3^: D_H_ and PI were measured in water by DLS; ^4^: ζ measures the electrophoretic mobility of NPs.

**Table 2:** Shell thickness of NPs and percentage of PEG on NPs surface.

Type	Shell Thickness ^1^ (nm)	Surface PEG ^2^ (wt %)
PEG_1K_-NPs	2.5 ± 0.4	3
PEG_2K_-NPs	3.4 ± 0.8	11
PEG_5K_-NPs	4.0 ± 0.2	5
Am/PEG_1K_-NPs	3.5 ± 0.2	2
Am/PEG_2K_-NPs	6.8 ± 1.2	4
Am/PEG_5K_-NPs	11.8 ± 0.9	2

^1^: Slope of the regression line of FALT analysis in Figure 3; ^2^: Surface PEG calculated by ^1^H NMR as described in 2.5.

## Supplementary Materials

The following are available online at https://www.mdpi.com/2079-4991/9/10/1354/s1, Supplementary information. Table S1: Concentration of NPs employed to study macrophage interaction, Table S2: Theoretical and experimental molecular weights of synthesized PEG-PCL copolymers, Table S3: Properties of FRET NPs, Figure S1: FTIR spectra of N_3_-PCL_4k_-N_3_ and H_2_N-PCL_4k_-NH_2_, Figure S2: NP stability over 24 h assessed through scattering of NPs sample at λ = 500 nm and representative size distribution curves of PEG-NPs and Am-NPs and Am/PEG-NPs, Figure S3: Scattering of NPs sample at λ = 500 nm in the presence of HSA or mucin, Figure S4: Fluorescence spectra of mucin (0.08%) in the presence of NPs (0.2 mg/mL), Figure S5: Stability of PEG-NPs or Amine-NPs and Amine/PEG-NPs in PBS at pH 7.4 along time, Figure S6: Stability of NPs in SILF at pH 7.4 up to 24 h, Figure S7: Emission spectra collected at λ_ex_ = 488 nm (DiO excitation) of PEG-NPs and Am-NPs and Am/PEG-NPs loaded with DiO/DiL permeated through an ECM gel and an artificial mucus, Figure S8: Confocal images of human monocytes after incubation with DiL-loaded NPs 50 μg/mL at 37 °C for 2 h.
