# Supplementary material for: Surface Exposure of PEG and Amines on Biodegradable Nanoparticles as a Strategy to Tune Their Interaction with Protein-Rich Biological Media
Source: Nanomaterials (Basel). 2019 Sep 20;9(10):1354. doi: 10.3390/nano9101354 (PMC6835417; doi:10.3390/nano9101354)
Supplement: Supplementary file 1 [file nanomaterials-09-01354-s001.pdf]

nanomaterials

Article

Surface Exposure of PEG and Amines on Biodegradable Nanoparticles as a Strategy to Tune Their Interaction with Protein-Rich Biological Media

Claudia Conte [1], Giovanni Dal Poggetto [2], Benjamin J. Swartzwelter [3], Diletta Esposito [1], Francesca Ungaro [1], Paola Laurienzo [2], Diana Boraschi [3,4] and Fabiana Quaglia [1]*

[1] Department of Pharmacy, University of Napoli Federico II, Via D. Montesano 49, 80131 Napoli, Italy; claudia.conte@unina.it (C.C.); diletta.esposito@unina.it (D.E.); ungaro@unina.it (F.U.)
[2] Institute for Polymers, Composites and Biomaterials, CNR, Via C. Flegrei 34, 80078 Pozzuoli (Napoli), Italy; giovanni.dalpoggetto@ipcb.cnr.it (G.D.P.); paola.laurienzo@ipcb.cnr.it (P.L.)
[3] Institute of Biochemistry and Cell Biology, CNR, Via P. Castellino 111, 80131 Napoli, Italy; swartzwe@colorado.edu (B.J.S.); d.boraschi@ibp.cnr.it (D.B.)
[4] Stazione Zoologica Anton Dohrn, Via A. Caracciolo 333, 80121 Napoli, Italy
* Correspondence: quaglia@unina.it (F.Q.)

Received: 19 August 2019; Accepted: 17 September 2019; Published: 20 September 2019

Preparation of media for transport experiments

Artificial mucus

For the experiment, 5 mL of artificial mucus (AM) were prepared by initially solubilising 20 mg of DNA from calf thymus in 3 mL of purified water followed by the addition of 25 µL of sterile egg yolk emulsion, 25 mg of type II porcine mucin, 30 µL of DPTA stock solution (1 mg/mL), 25 mg NaCl, 11 mg KCl and 0.1 mL of RPMI. The volume was made up to 5 mL with purified water and allowed to mix until a cloudy uniform dispersion was obtained [16].

Simulated Interstitial Lung Fluid

A 100 mL aliquot of Simulated Interstitial Lung Fluid (SILF) contains 600 mg of NaCl, 30 mg of KCl, 15.7 mg of disodium phosphate (Na_2HPO_4), 7.1 mg of sodium sulfate (Na_2SO_4), 27.1 mg of calcium chloride ($CaCl_2$), 57.4 mg of sodium acetate ($NaH_3C_2O_2$), 260.2 mg of sodium bicarbonate ($NaHCO_3$), 9.8 mg of sodium citrate dihydrate and 29.4 mg of hydrated magnesium chloride ($MgCl_2.6H_2O$).

Table S1. Concentration of NPs employed to study macrophage interaction. The calculation accounted for the volume and surface area of each type of particle.

Samples	NP size (nm)	Conc. (µg/mL)
PEG_{1k}-NPs	70	25.0
PEG_{2k}-NPs	45	16.1
PEG_{5k}-NPs	45	16.1
Am-NPs	130	46.4
Am/PEG_{1k}-NPs	120	42.9
Am/PEG_{2k}-NPs	100	35.7
Am/PEG_{5k}-NPs	100	35.7

Table S2. Theoretical and experimental molecular weights of synthesized PEG-PCL copolymers.

Copolymer	Mn (Da)[1] (theoretical)	Mn (Da)[1] (experimental)	Mn (Da)[2]	Mw (Da)[2]	PDI[2] (Mw/Mn)
$mPEG_{1k}$-PCL_{4k}	5104	5446	5059	6737	1.32
$mPEG_{2k}$-PCL_{4k}	6104	5789	6194	8420	1.35

[1] Calculated or experimentally evaluated on the basis of OH/ε-CL molar ratio (Mn =114,14 x ([ε-CL]/[mPEG]) x conversion ε-CL + Mn of mPEG); [2] Determined by GPC.

Table S3. Properties of FRET NPs.

Type	DH (nm±SD)	PI	ζ (mV±SD)	Encaps. eff DiO (%)	Encaps. eff DiL (%)
PEG_{1K}-NPs	165.8 ± 11.9	0.249	-8.3±2.0	70	62
PEG_{2K}-NPs	95.8 ± 26.2	0.410	-15.3±2.7	76	55
PEG_{5K}-NPs	124.2 ± 19.4	0.257	-9.4±0.7	100	100
Am-NPs	134.4±0.3	0.128	34.3±1.3	81	83
Am/PEG_{1K}-NPs	137.1 ± 6.7	0.109	30.1±1.3	62	83
Am/PEG_{2K}-NPs	131.8 ± 22.6	0.158	29.5±4.1	83	100
Am/PEG_{5K}-NPs	112.3 ± 1.6	0.154	22.1±3.2	70	100

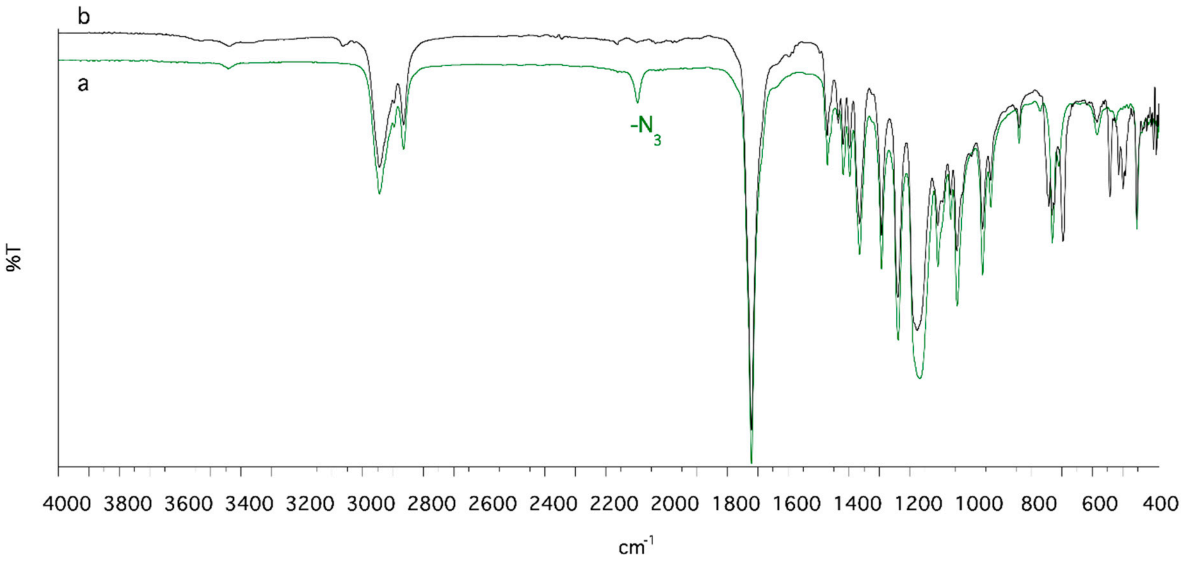

Figure S1. FTIR spectra of N_3-PCL_{4k}-N_3 (a) and H_2N-PCL_{4k}-NH_2 (b). Spectra were acquired at a resolution of 2 cm^{-1} (average of 20 scans).

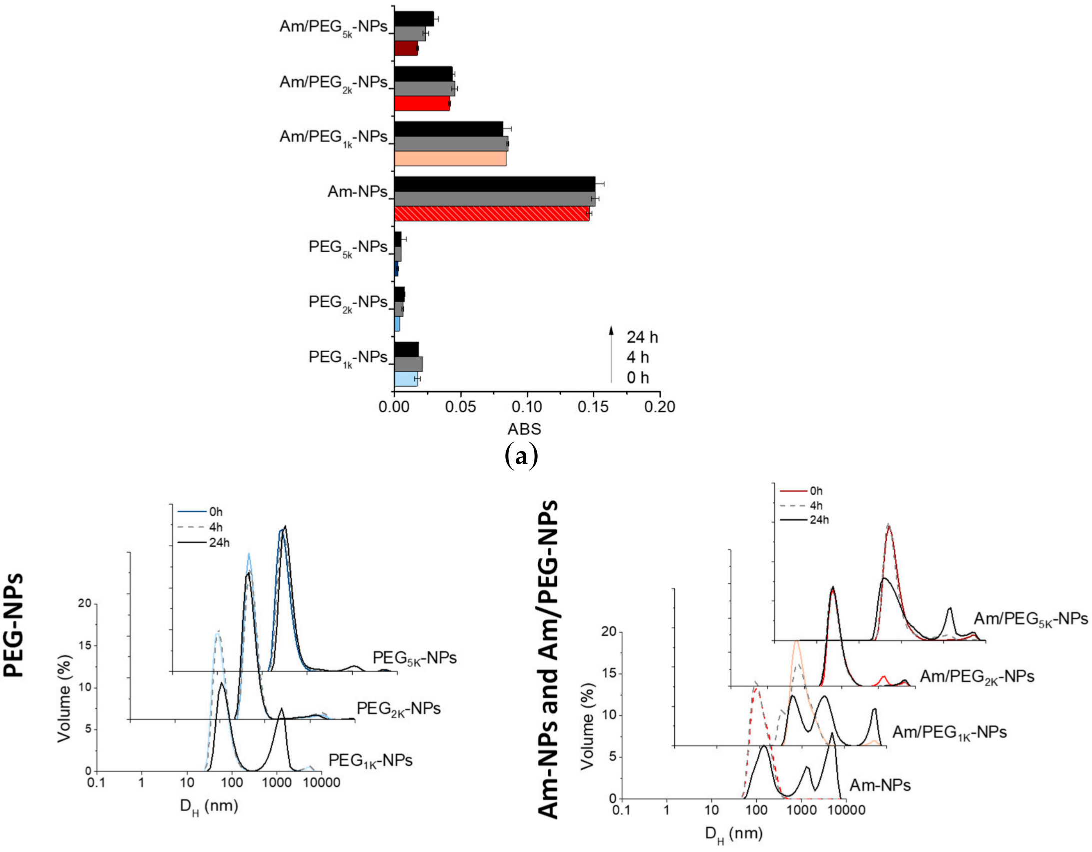

(**a**)

(**b**) (**c**)

Figure S2. NP stability over 24 h assessed through (**a**) scattering of NPs sample at λ=500 nm and representative size distribution curves of PEG-NPs (**b**) and Am-NPs and Am/PEG-NPs (**c**). NP concentration was 0.5 mg/mL. Data are reported as an average of duplicate experiments ± SD.

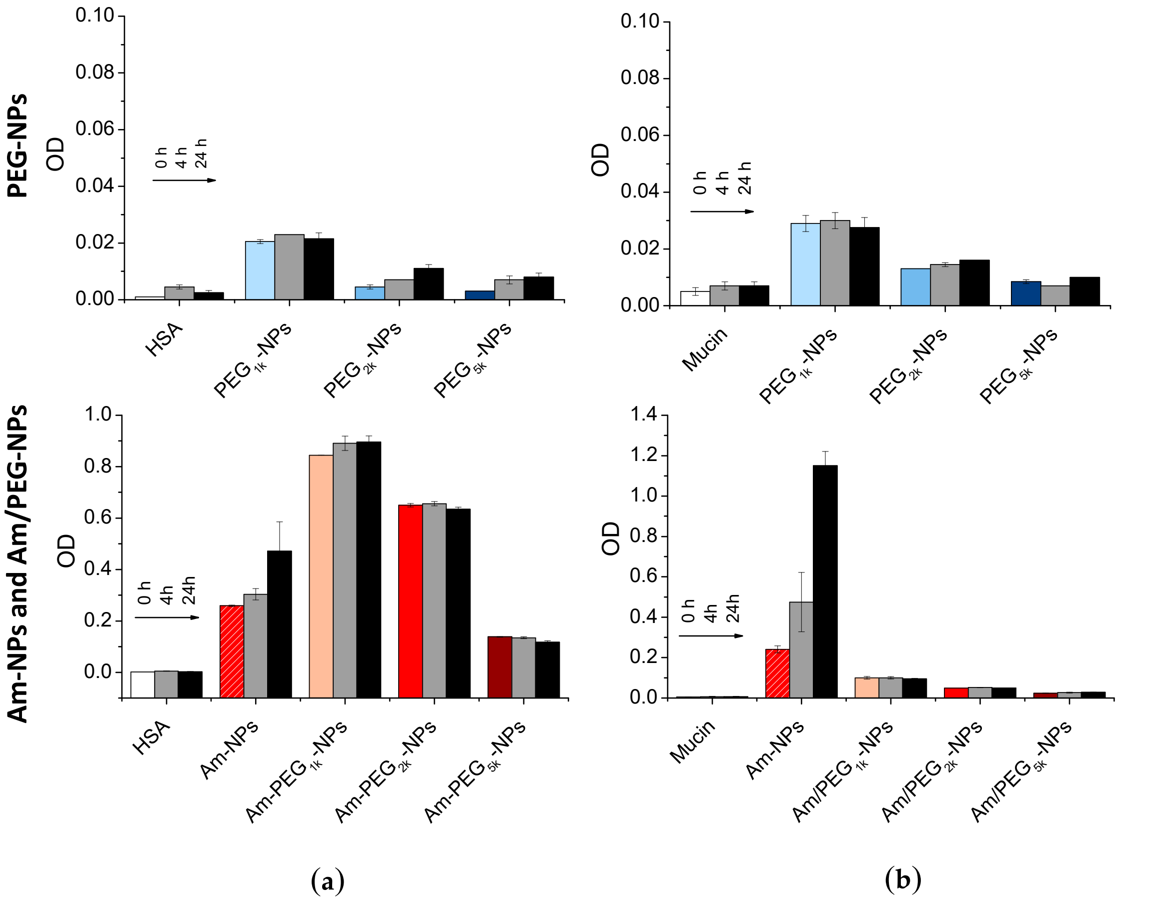

Figure S3. Scattering of NPs sample at λ=500 nm in the presence of **a**) HSA or **b**) mucin. PEG-NPs (top); Am-NPs and Am/PEG-NPs (bottom). NPs=0.5 mg/mL, HSA=0.2 mg/mL, mucin=0.08% w/w. Data are reported as an average of duplicate experiments ± SD. .

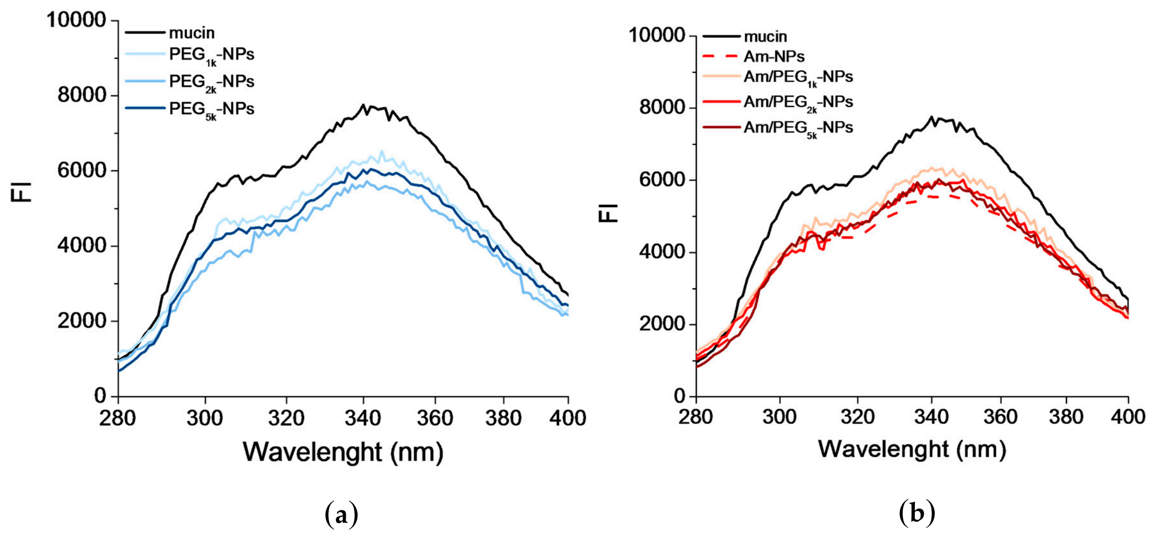

Figure S4. Fluorescence spectra of mucin (0.08%) in the presence of NPs (0.2 mg/mL); (**a**) PEG-NPs and (**b**) Am-NPs and Am/PEG-NPs. Spectrum of free mucin is reported as control.

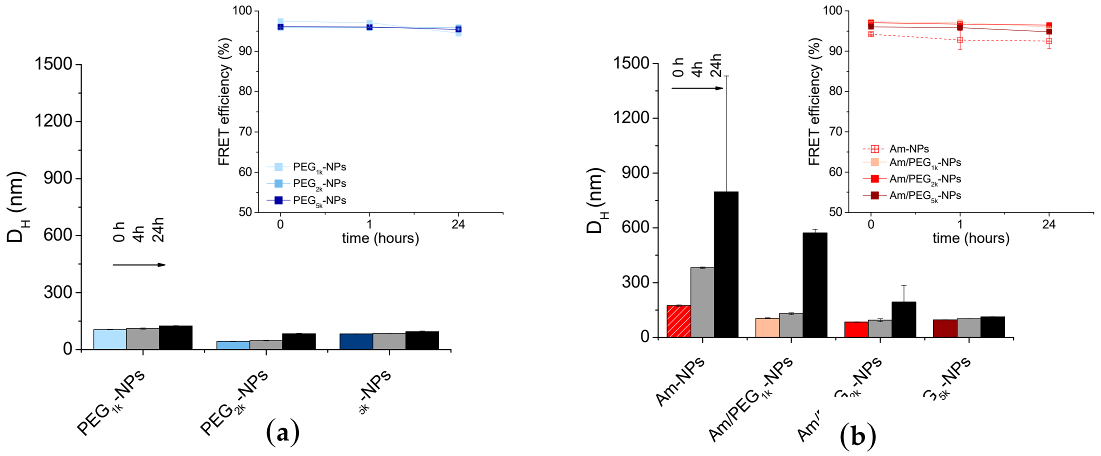

Figure S5. Stability of PEG-NPs (**a**) or Amine-NPs and Amine/PEG-NPs (**b**) in PBS at pH 7.4 along time. In the inset FRET efficiency of NPs along time is reported. NP concentration was 0.5 mg/mL. Data are reported as an average of duplicate experiments ± SD. .

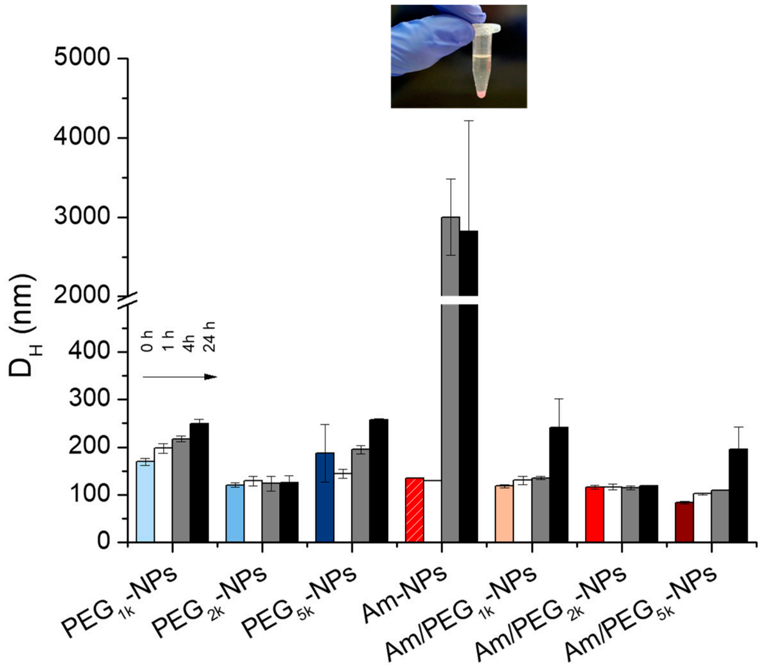

Figure S6. Stability of NPs in SILF at pH 7.4 up to 24 h. NP concentration was 0.5 mg/mL. Data are reported as an average of duplicate experiments ± SD.

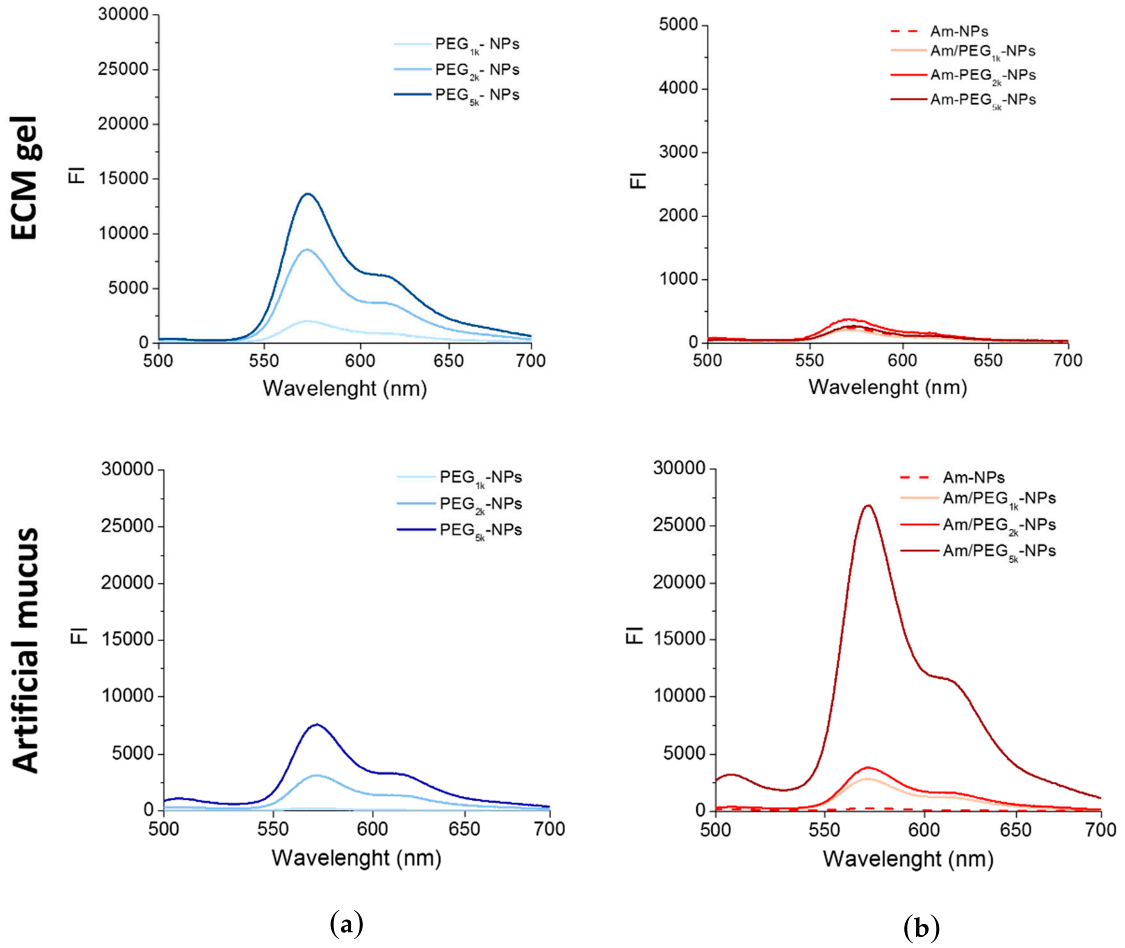

Figure S7. Emission spectra collected at λ_{ex} = 488 nm (DiO excitation) of PEG-NPs (**a**) and Am-NPs and Am/PEG-NPs (**b**) loaded with DiO/DiL permeated through an ECM gel and an artificial mucus.

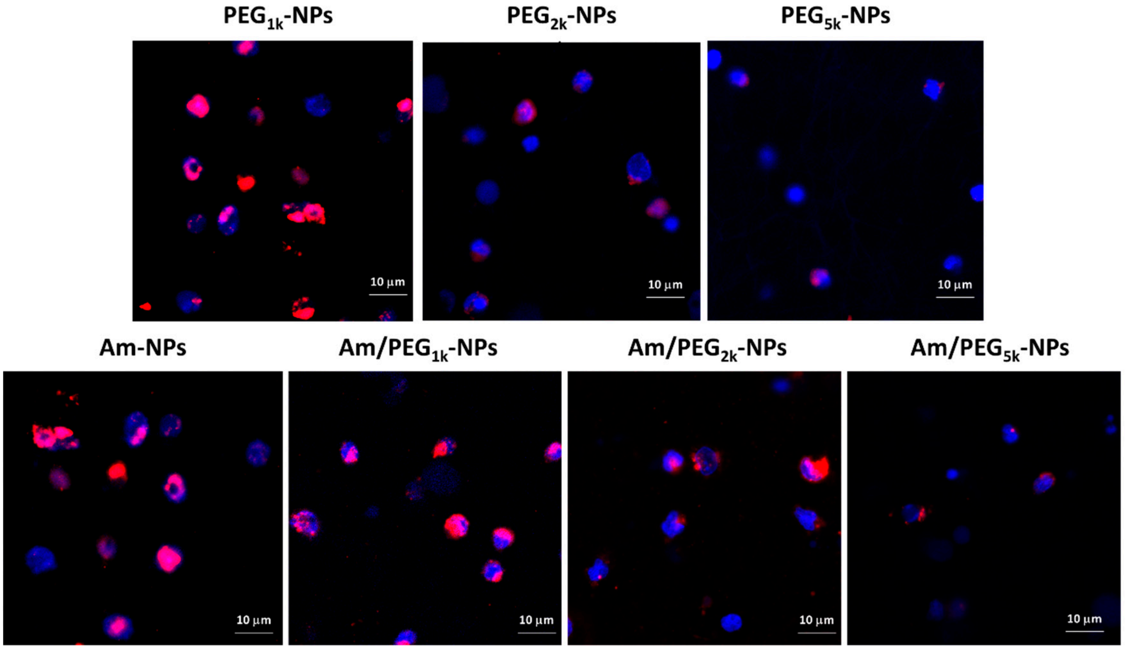

Figure S8. Confocal images of human monocytes after incubation with DiL-loaded NPs 50   g/mL at 37°C for 2 h. Images were acquired at λex = 543 nm and spectral filter LP 560 nm for DiL detection.

Zen 2009 image Software was used for image processing. Blue, DAPI-stained cell nuclei; red, DiL showing the NP uptake.

© 2019 by the authors. Submitted for possible open access publication under the terms and conditions of the Creative Commons Attribution (CC BY) license (http://creativecommons.org/licenses/by/4.0/).
